# Generation of Multiple‐Depth 3D Computer‐Generated Holograms from 2D‐Image‐Datasets Trained CNN

**DOI:** 10.1002/advs.202408610

**Published:** 2024-12-31

**Authors:** Xingpeng Yan, Jiaqi Li, Yanan Zhang, Hebin Chang, Hairong Hu, Tao Jing, Hanyu Li, Yang Zhang, Jinhong Xue, Xunbo Yu, Xiaoyu Jiang

**Affiliations:** ^1^ Department of Information Communication Army Academy of Armored Forces Beijing 100072 China; ^2^ School of Electronic Engineering Beijing University of Posts and Telecommunications Beijing 100080 China; ^3^ Department of Mechanical Engineering Army Engineering University of PLA Nanjing 210007 China

**Keywords:** CNN, computer‐generated hologram, virtual depth datasets

## Abstract

Generating computer‐generated holograms (CGHs) for 3D scenes by learning‐based methods can reconstruct arbitrary 3D scenes with higher quality and faster speed. However, the homogenization and difficulty of obtaining 3D high‐resolution datasets seriously limit the generalization ability of the model. A novel approach is proposed to train 3D encoding models based on convolutional neural networks (CNNs) using 2D image datasets. This technique produces virtual depth (VD) images with a statistically uniform distribution. This approach employs a CNN trained with the angular spectrum method (ASM) for calculating diffraction fields layer by layer. A fully convolutional neural network architecture for phase‐only encoding, which is trained on the DIV2K‐VD dataset. Experimental results validate its effectiveness by generating a 4K phase‐only hologram within only 0.061 s, yielding high‐quality holograms that have an average PSNR of 34.7 dB along with an SSIM of 0.836, offering high quality, economic and time efficiencies compared to traditional methods.

## Graphical abstract

This study presents a novel method for generating computer‐generated holograms (CGHs) of 3D scenes using a CNN trained on 2D image datasets. By creating virtual depth (VD) images with a uniform statistical distribution, this approach enables fast reconstructions, achieving phase‐only holograms with an average PSNR of 34.7 dB and SSIM of 0.836, surpassing traditional techniques.

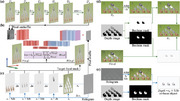

## 1 Introduction

Holographic 3D display technology, encompassing interference recording and diffraction reproduction, captures complete optical field information.^[^ ^1^ ^]^ This capability provides comprehensive visual cues, including focusing, interocular disparity, and motion parallax, with significant applications in fields such as medical treatment, industrial inspection, education, and entertainment.^[^ ^2^, ^3^, ^4^, ^5^, ^6^, ^7^ ^]^ This authentic 3D display concept was originally proposed by Dennis Gabor.^[^ ^8^ ^]^ Although, traditional optical holography necessitates complex steps like development and drying, the advent of computer‐generated holograms (CGH) has addressed these limitations by delivering precise depth information without the need for intricate recording paths.^[^ ^9^ ^]^ CGH, facilitated by spatial light modulators (SLM), allows for dynamic real‐time holographic displays,^[^ ^10^, ^11^, ^12^, ^13^ ^]^ enhancing user experience while mitigating visual fatigue associated with focus adjustment.

CGH can be classified according to the types of information it contains, specifically into phase‐only CGH, amplitude‐only CGH, and complex‐amplitude CGH.^[^ ^14^, ^15^, ^16^ ^]^ Phase‐only CGH replicates the propagation of light from an object scene, often without the necessity for reference light, by converting complex amplitude information in the diffracted field directly into phase information. This method has been chosen as the central focus of our study due to its higher diffraction efficiency and wider applicability.^[^ ^17^, ^18^ ^]^ Regarding the representation of basic elements in CGH, simulation‐based methods depict the scene through point clouds,^[^ ^19^ ^]^ light fields,^[^ ^20^, ^21^ ^]^ polygon meshes,^[^ ^22^, ^23^ ^]^ RGB‐D images,^[^ ^24^ ^]^ or multi‐layer images.^[^ ^24^, ^25^ ^]^ These representations are used to numerically simulate diffraction and interference via techniques such as the angular spectrum method^[^ ^26^ ^]^ or Kirchhoff/Fresnel diffraction. The encoding of CGH can be framed as an inverse problem, which is addressed using optimization algorithms such as iterative phase‐retrieval methods^[^ ^27^, ^28^ ^]^ and (stochastic) gradient descent.^[^ ^29^, ^30^ ^]^ The integration of neural networks into this framework allows for the approximation of complex mappings, thereby enhancing holographic quality and processing speed, and resolving the historical trade‐offs between reproduction quality and computational efficiency.

The application of neural networks in phase hologram generation has evolved significantly since its inception in 1998, when Yamauchi proposed an early algorithm.^[^ ^31^ ^]^ A notable advancement occurred in 2018, when Horisaki et al. utilized deep learning to generate phase holograms, employing a residual CNN that has since become prevalent in computational optics.^[^ ^32^, ^33^, ^34^ ^]^ This approach drastically reduced generation times to milliseconds; however, further improvement is needed for performance on real images. In response, Lee et al. refined the dataset in 2020, utilizing 10 000 varied circular images, which markedly enhanced hologram quality.^[^ ^35^ ^]^ In 2021, Shi et al. introduced the TensorHolo network, achieving real‐time 2K hologram generation on smartphones.^[^ ^36^ ^]^ Their 2022 TensorHolo V2 further optimized the process by facilitating end‐to‐end training for image‐to‐phase hologram conversion.^[^ ^37^ ^]^ Additionally, data‐driven deep learning has been applied to various holographic tasks, including aberration correction,^[^ ^38^ ^]^ hologram compression,^[^ ^39^, ^40^ ^]^ and super‐resolution.^[^ ^41^ ^]^ Moreover, HoloNet–a non‐end‐to‐end framework introduced by Peng et al. in 2020–dissects the generation process using two neural networks.^[^ ^42^, ^43^ ^]^ Wu et al. later proposed Holo‐Encoder in 2021, which predicts holograms from input images using a single end‐to‐end model.^[^ ^44^ ^]^ Recent work by Liu et al. in 2022 incorporated 2D phase grating considerations into the modeling process, while 2023 saw the introduction of 4K‐DMDNet, further enhancing diffraction model networks.^[^ ^45^, ^46^ ^]^ In 2024, Zhong et al. introduce the CCNN‐CGH, a groundbreaking complex‐valued neural network architecture that transcends the inefficiencies of traditional real‐valued networks in holography. By harnessing the complex amplitude properties inherent in light fields, the CCNN‐CGH achieves enhanced representational capacity, resulting in superior efficiency and quality for CGH generation.^[^ ^47^ ^]^

Neural network‐based CGH training heavily relies on datasets. The generation of CGH using neural networks trained on 2D datasets has seen significant advancements. In 2018, Horisaki et al. employed a deep ResNet to calculate holograms,^[^ ^32^ ^]^ utilizing a training dataset comprised of uniform random phase patterns and their Fresnel propagating patterns, totaling 100,000 speckle pairs. The pixel sizes for both the holograms and target patterns were set at 16 × 16, 32 × 32, and 64 × 64. Subsequently, in 2021, Horisaki et al. built upon the ResNet architecture to propose a non‐iterative 3D CGH method, which aimed to reproduce a 3D intensity pattern from handwritten digits sourced from the 28 × 28 EMNIST dataset.^[^ ^48^ ^]^ In 2020, Khan et al. introduced a GAN‐based DNN for holography,^[^ ^49^ ^]^ also utilizing the EMNIST dataset alongside its corresponding holograms. Aiming for spatially varying aberration compensation, Yoo et al. integrated an FFT‐based convolution with a neural network,^[^ ^38^ ^]^ leveraging pairs of DIV2K images and aberration compensation maps. Wu et al. developed the Holo‐Encoder, an autoencoder‐based neural network for phase‐only hologram generation, which incorporated the Fresnel diffraction model for unsupervised training using the DIV2K dataset.^[^ ^44^ ^]^ Wang et al. (2022) proposed a real‐time method for generating phase‐only holograms with a CNN trained on a Low‐Frequency Mixed Noise (LFMN) dataset, showcasing significant advantages over conventional methods.^[^ ^34^ ^]^ The DIV2K dataset, a large super‐resolution reconstruction dataset with 1000 high‐definition 2K images, has proven essential in avoiding overfitting in learning‐based CGH tasks.^[^ ^38^, ^44^ ^]^

The generation of CGH with neural networks trained on 3D datasets faces challenges due to the scarcity of high‐quality 3D graphical training datasets, which require substantial temporal, human, and financial resources. In 2012, Silberman et al. captured real‐world scene intensity and depth data using a depth camera, contributing valuable insights for depth perception and scene segmentation.^[^ ^50^ ^]^ The collection of real‐world 3D datasets typically involves costly and time‐consuming equipment such as laser scanners. For instance, time‐of‐flight (TOF) depth cameras, which include multiple sophisticated components, are known for their high costs and susceptibility to multiple reflections.^[^ ^51^ ^]^ In 2021, Shi et al. utilized the MIT‐CGH‐4K dataset, which features 4000 RGB‐D images with pixel sizes of 192 × 192 and 384 × 384 generated by a 3D random scene generator.^[^ ^36^ ^]^ This software‐based approach offers convenience compared to traditional methods but presents challenges in scene setup and high‐performance GPU reliance. In 2022, Shi et al. advanced this field by introducing the MIT‐CGH‐4K‐V2 dataset, which employed Layered Depth Images (LDI) to enhance computational efficiency and data performance.^[^ ^37^ ^]^ Their work, integrating supervised and unsupervised learning to synthesize high‐quality 3D phase‐only holograms, significantly improved the real‐time capabilities and image quality of holographic displays. More recently, Liu et al. (2024) used 3D modeling software to acquire RGB‐D datasets at 2160 × 3840 pixels, while Wang et al. (2024) developed a holographic camera that captures high‐fidelity holograms of real 3D scenes through an electrically tunable liquid lens and EEPMD‐Net, enabling accurate holographic reproduction.^[^ ^52^ ^]^

In the diffraction encoding process of 3D computational holography, neural network encodes complex amplitudes into a phase‐only hologram. The input data, comprising spatial complex signals such as intensity and depth images (RGB‐D), simulate the complex amplitude of the diffraction field via computational methods. The input are then transformed by the neural network into phase signals, encoding phase‐only holograms. The loss function is defined as the difference between the scene's intensity information and the reconstructed 3D scene's stacked intensity information from the hologram.

The distribution of complex amplitudes of a 3D scene's diffraction field is determined by its intensity and depth distributions. Different intensity and depth distributions produce varied complex amplitude distributions. To effectively ensure the neural network model's encoding and generalization abilities, a 3D dataset should cover diverse intensity distributions across all depth layers. However, constructing such a dataset is challenging because it necessitates addressing both the diversity of intensity distributions and the uniformity of depth distributions, as exemplified by the complexity and importance of the MIT dataset.^[^ ^36^ ^]^ Thus, the quality of the 3D dataset, determined by the distributions of intensity and depth, is crucial for training the neural network's hologram encoding capability.

It is noteworthy that the neural network's encoding ability is closely related to the distributions of intensity and depth but not to the correspondence between intensity and depth, which might be counterintuitive. Furthermore, different intensity distributions have different spectra, the depth distribution affects only the maximum sampling frequency. For sampled 2D image datasets, the depth distribution does not impact the spectral distribution itself. Thus, a 2D image dataset with uniformly distributed depths and diverse intensity distributions can ensure the neural network model's encoding and generalization capabilities.

This research utilizes the DIV2K dataset and applies the random mapping and resampling method (RMRM) to assign virtual depth (VD) values to the DIV2K dataset, resulting in a uniformly distributed VD despite its inherent inaccuracies. Then, a neural network is trained to generate multi‐depth holograms using the angular spectrum method (ASM) to compute diffraction fields layer by layer. A fully convolutional neural network based on CNN architecture is employed for phase‐only encoding of the complex amplitudes. To fully appreciate this counterintuitive approach, it is necessary to understand the detailed encoding training process of the CNN.

## 2 CNN‐Based Neural Network for Generating Multi‐Depth Phase‐Only Holograms

**Figure 1:**
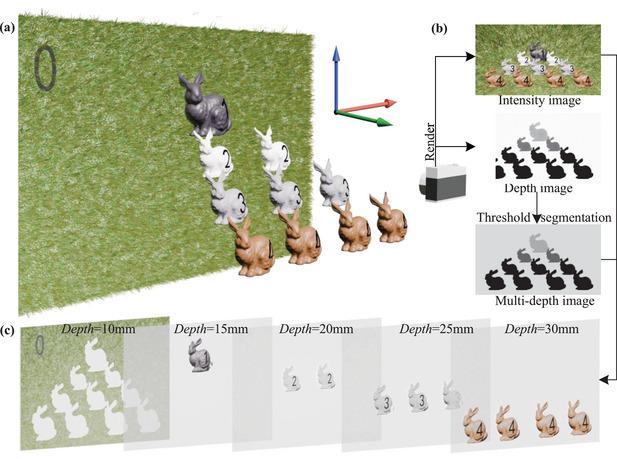
Generation of multi‐depth scene. a) 3D scene, b) intensity image, depth image and multi‐depth image, c) multi‐depth scene.

For ease of description, this paper introduces a 3D scene as illustrated in **Figure** **1**a. The scene, when sampled, yields intensity images as depicted in Figure 1b, while depth sampling produces grayscale depth images where depth is represented by shades, as shown in Figure 1b. As presented in Figure 1c, the depth range of the scene spans from 10 to 30 mm, with the hologram plane situated at a depth of 0 mm. The scene is segmented into 5 depth layers on average. This depth range was chosen to align with near‐eye display applications, where precise depth segmentation is crucial for achieving comfortable stereoscopic vision. A smaller depth interval ensures smoother transitions between layers but increases the computational load, which is constrained by current hardware capabilities. In fact, the method can be extended for application when the number of layers is greater than or equal to 2. While our current implementation utilizes five depth layers to maintain computational efficiency, we acknowledge the importance of exploring larger numbers of depth layers for enhanced human visual perception. Recent advancements, such as those reported by Di Wang et al. (2024) in their work on decimeter‐depth and polarization addressable color 3D meta‐holography, demonstrate the potential of employing advanced techniques, including holographic multiplexing and metasurface design, to optimize phase information and address the limitations of large depth imaging.^[^ ^53^ ^]^ The annotated elements include a grassy background labeled as 0, positioned at *Depth* = 10 mm, a black rabbit labeled as 1 at *Depth* = 15 mm, a white rabbit labeled as 2 at *Depth* = 20 mm, a rabbit labeled as 3 at *Depth* = 25 mm, and a rabbit labeled as 4 at *Depth* = 30 mm.

**Figure 2:**
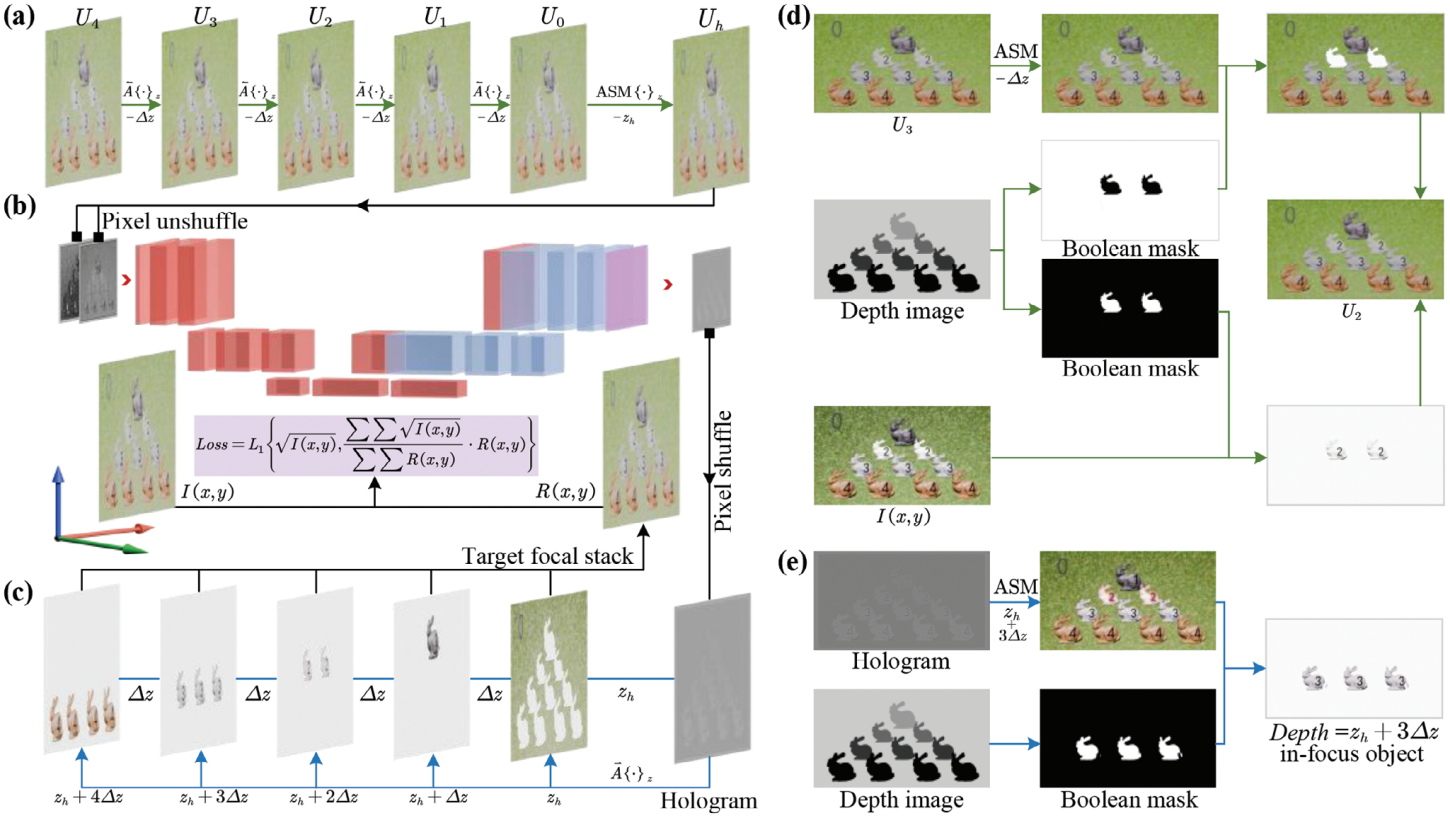
Training of CNN‐Based neural network model for generating multi‐depth phase‐only holograms. a) Calculating inverse diffraction field, b) encoding phase‐only holograms with CNN, c) obtain the target focal stack by reconstructing multi‐depth in‐focus object,^[^ ^36^ ^]^ d) $\overset{↼}{A}{\left\{\cdot \right\}}_{z}$ operation, using *U*_3_ to calculate *U*_2_ as an example, e) $\vec{A}{\left\{\cdot \right\}}_{z}$ operation, take the example of computing the in‐focus object when *Depth* = *z*_*h*_ + 3Δ*z*.

For the sake of simplicity, this paper exclusively focuses on the diffraction recording and reproduction in the grayscale channel, with the understanding that the principles similarly apply to the RGB channels. The training process of the model‐driven neural network employed in this study, as depicted in **Figure** **2**, comprises three key stages: computation of layered inverse diffraction fields, holographic reconstruction of multi‐depth diffraction fields and encoding phase‐only holograms with CNN.

### 2.1 Computation of Layered Inverse Diffraction Fields

This layer‐by‐layer replacement method (L2RM) replaces the depth intensity image of the first layer with the complete intensity image complex amplitude, achieving the removal of occluding black lines. This approach not only avoids increasing computational complexity but also effectively enhances the quality of holographic image reproduction.^[^ ^18^ ^]^

The computational process is illustrated in Figure 2a,d, where the layered diffraction process is consistent for the first four depth layers. Taking the computation process in Figure 2d as an example, the complex amplitude *U*_3_(*x*, *y*) when *Depth* = *z*_*h*_ + 3Δ*z*, intensity image *I*(*x*, *y*), and depth image *D*(*x*, *y*) are known, the objective is to determine the complex amplitude *U*_2_(*x*, *y*) when *Depth* = *z*_*h*_ + 2Δ*z*. In this study, the initial phase is set to zero instead of being randomly initialized. However, we clarify that this choice was made intentionally; each depth plane of the multi‐depth scene is initialized with phase values corresponding to integer multiples of the wavelength. This approach allows for uniform phase initialization across all depth planes, ensuring coherent wavefront propagation, while setting the phase to zero is simply one of several valid options. This deterministic phase initialization method is critical to the success of CNN training, as it ensures the complex holograms generated for the entire dataset are statistically consistent and bear repetitive features that can be learned by a CNN.^[^ ^36^ ^]^

First, the complex amplitude *U*_3_(*x*, *y*) for *Depth* = *z*_*h*_ + 3Δ*z* is obtained by diffracting at a distance of −Δ*z* using ASM. The diffracted field 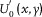 is then combined with the amplitude of the intensity image $\sqrt{I\left(x,y\right)}$ and the Boolean mask *M*_3_(*x*, *y*) through a dot product, yielding the complex amplitude *U*_2_(*x*, *y*) for *Depth* = *z*_*h*_ + 2Δ*z*. Upon acquiring the complex amplitude *U*_0_(*x*, *y*) for *Depth* = *z*_*h*_, the complex amplitude *U*_*h*_(*x*, *y*) for the hologram plane is obtained by diffracting at a distance of −*z*_*h*_ using ASM.

The first four iterations of the hierarchical diffraction process can be extended to a more general scenario without loss of generality. Assuming the scene is uniformly divided into *N* depths with a spacing of Δ*z*, according to the angular spectrum theory, the complex amplitude calculation process using the ASM is expressed as follows:

(1) $$\begin{array}{ccc} U_{n-1}\left(x,y\right) & = & \overset{↼}{A}{\left\{U_{n}\left(x,y\right)\right\}}_{z=-\Delta z}\\ & = & \sqrt{I(x,y)}\cdot M_{n}\left(x,y\right)+\text{ASM}{\left\{U_{n}\left(x,y\right)\right\}}_{z=-\Delta z}\\ & & \cdot (1-M_{n}\left(x,y\right)) \end{array}$$

where, $\overset{↼}{A}{\left\{\cdot \right\}}_{z=-\Delta z}$ denotes the reverse diffraction process with a diffraction distance of −Δ*z*. The term $\sqrt{I\left(x,y\right)}$ represents the amplitude of the intensity image, and *M*_*n*_(*x*, *y*) corresponds to the Boolean mask for *Depth* = *z*_*h*_ + *n*Δ*z*. The notation ASM {·}_*z* = −Δ*z*_ signifies the ASM of diffraction. The calculation method can be expressed as follows:

(2) $$\begin{array}{c} \text{ASM}{\left\{U_{n}\left(x,y\right)\right\}}_{z=-\Delta z}=F^{-1}\left\{F\left\{U_{n}\left(x,y\right)\right\}\cdot H_{-\Delta z}\left(f_{x},f_{y}\right)\right\} \end{array}$$

where, *F*{·} is the 2D Fourier transform while *F* ^−1^{·} is the 2D inverse Fourier transform, and *H*_−Δ*z*_(*f*_*x*_, *f*_*y*_) is the transfer function, the calculation method can be expressed as follows:

(3) $$\begin{array}{c} H_{-\Delta z}\left(f_{x},f_{y}\right)=\exp\left[jk\left(-\Delta z\right)\sqrt{1-{\left(\lambda f_{x}\right)}^{2}-{\left(\lambda f_{y}\right)}^{2}}\right] \end{array}$$

where, j represents the imaginary unit, where j^2^ = −1. The symbol λ denotes the wavelength of the laser, and $k=\frac{2\pi }{\lambda }$ is the wave number. The conditions $|f_{x}\left|,\right|f_{y}|<\frac{1}{2\Delta p}$ hold, with Δ*p* representing the pixel pitch of the SLM. The complex amplitude *U*_*h*_(*x*, *y*) of a holographic planar display at specific reconstruction depths can be expressed as follows:

(4) $$\begin{array}{c} U_{h}\left(x,y\right)=\text{ASM}{\left\{U_{0}\left(x,y\right)\right\}}_{z=-z_{h}} \end{array}$$

where, the term −*z*_*h*_ denotes the retrograde distance from the depth layer *Depth* = *z*_*h*_ to the holographic plane *Depth* = 0.

The complex amplitude on the plane where the SLM is situated can be obtained using the above‐mentioned method. With the aid of a neural network, this complex amplitude is then utilized to encode and generate holograms with multiple depths, denoted as *U*_*h*_(*x*, *y*).

### 2.2 Holographic Reconstruction of Multi‐Depth Diffraction Fields

For the sake of clarity, we first describe the holographic diffraction reconstruction process, as illustrated in Figure 2c. Phase‐only holograms undergo diffraction reconstruction at different depths. The reconstructed focused light fields at different focal planes can be obtained by taking the dot product with a binary mask. Subsequently, the superimposed reconstructed light field is obtained. Taking the focused light field at *Depth* = *z*_*h*_ + 3Δ*z* as an example, as shown in Figure 2e, the hologram undergoes forward diffraction propagation using ASM over a distance of $z_{h}+3\Delta z$, resulting in the reconstructed light field. In this case, the three rabbits at depth 3 are focused on their corresponding focal plane, while other positions appear blurred. The in‐focus pixel values at the *Depth* = *z*_*h*_ + 3Δ*z* focal plane are then obtained by taking the dot product with a binary mask.

The process of obtaining the reconstructed focused light field *R*_*m*_(*x*, *y*) at different focal planes can also be expressed as follows:

(5) $$\begin{array}{cc} R_{m}\left(x,y\right) & =\left|\vec{A}{\left\{U_{H}\left(x,y\right)\right\}}_{z=z_{h}+m\Delta z}\right|\\ & =\left|\text{ASM}{\left\{U_{H}\left(x,y\right)\right\}}_{z=z_{h}+m\Delta z}\cdot M_{m}\left(x,y\right)\right| \end{array}$$

where, the notation *N* denotes the number of depth layers of the multi‐depth scene, *m* = *N* − *n*, the notation $\vec{A}{\left\{\cdot \right\}}_{z=z_{h}+m\Delta z}$ signifies the forward diffraction process, reproducing the distance $\left(z_{h}+n\Delta z\right)$, with *U*_*H*_(*x*, *y*) representing the complex amplitude of the hologram, and *M*_*m*_(*x*, *y*) indicating the Boolean mask corresponding to the *Depth* = *z*_*h*_ + *m*Δ*z* plane. The term $\text{ASM}{\left\{\cdot \right\}}_{z=z_{h}+m\Delta z}$ denotes the ASM of diffraction, and its calculation method is expressed as follows:

(6) $$\begin{array}{c} \text{ASM}{\left\{U_{H}\left(x,y\right)\right\}}_{z=z_{h}+m\Delta z}=F^{-1}\left\{F\left\{U_{H}\left(x,y\right)\right\}\cdot H_{z=z_{h}+m\Delta z}\left(f_{x},f_{y}\right)\right\} \end{array}$$

By employing the aforementioned method, we can obtain the focused light field at each focal plane *R*_*m*_(*x*, *y*). Through superposition, the reconstructed target focal stack light field *R*(*x*, *y*) can be derived, and its calculation method is expressed as follows:

(7) $$\begin{array}{c} R\left(x,y\right)=\sum_{m=0}^{N}R_{m}\left(x,y\right) \end{array}$$

### 2.3 Encoding Phase‐Only Holograms with CNN

**Figure 3:**
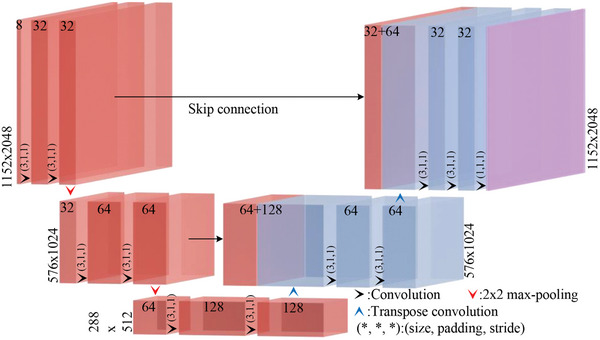
The structure of the CNN‐based neural network.

A fully convolutional neural network based on CNN is designed to encode phase‐only holograms, as illustrated in **Figure** **3**.

Inputs of the neural network. CNN derived from the complex amplitude *U*_*h*_(*x*, *y*), obtained through (2.1). To mitigate diffraction‐induced fringe patterns at the edges, zero‐padding is applied, expanding the resolution from 2160 × 3840 to 2304 × 4096. To facilitate holographic phase encoding, Euler's formula is utilized to convert the complex amplitude into two channels representing the real and imaginary parts. The operations “shuffle” and “unshuffle” are employed to improve model efficiency and generalization. These operations are applied to the data of the two channels, resulting in eight channels. This transformation yields a matrix of size 1152 × 2048 × 8, which serves as the input for the CNN. By redistributing adjacent spatial pixel values into separate channels, the “shuffle” operation enhances the resolution of feature extraction while reducing computational burden. The corresponding “unshuffle” process recombines these separated pixels, ensuring spatial consistency in the resulting hologram.

Outputs of the neural network. The output consists of a phase matrix with dimensions 1152 × 2048 × 4. By employing “phase recombination”, which utilizes the “pixel shuffle” technique as illustrated in Figure 2b, multiple‐depth holograms are obtained from the CNN output. The final phase displayed on the SLM is derived by interpreting the four‐channel phase matrix into a single phase distribution, ensuring that each holographic depth is accurately represented through precise pixel mapping and recombination.

Loss function of the neural network. The L1 (mean absolute error) loss function is utilized. Through a holographic reproduction process with a hologram of (2.2), the superimposed reproduced light field *R*(*x*, *y*) is generated. The residual, calculated as the difference between this reproduction and the amplitude $\sqrt{I\left(x,y\right)}$ of the ground truth amplitude, serves as the loss function. This facilitates the update of the network model parameters. The loss function can be expressed as follows:

(8) $$Loss=L_{1}\left\{\sqrt{I\left(x,y\right)},\frac{\sum \sum \cdot \sqrt{I\left(x,y\right)}}{\sum \sum R\left(x,y\right)}\cdot R\left(x,y\right)\right\}$$

where, the expression $\frac{\sum \sum \cdot \sqrt{I\left(x,y\right)}}{\sum \sum R\left(x,y\right)}\cdot R\left(x,y\right)$ represents a linear transformation designed to match the brightness of the ground truth intensity image. This adjustment can be achieved in optical experiments by modulating the laser power. Although photodetectors sample intensity rather than amplitude, our approach leverages the linear relationship between amplitude and intensity to maintain accuracy. The chosen loss function, which computes the mean absolute error between the original amplitude and the reconstructed amplitude, is robust due to this linearity, mitigating concerns about potential additional errors.

Architectures of the neural network. CNN consists of five convolutional modules with convolutional kernels (size, padding, stride) set to (3, 1, 1), two 2 × 2 max‐pooling downsampling modules, and two (2, 0, 4) transposed convolutional upsampling modules. The same‐sized convolutional modules on the left and right sides are connected by skip connections to facilitate the transfer of feature maps.

The residual of the input RGB image intensity is employed as the loss function. The CNN‐based model undergoes gradient descent backpropagation to adjust its parameters, optimizing the quality of encoding phase‐only holograms.

During the training process, depth images serve two functions. First, they segment the amplitude before inputting it into the CNN, enabling the layer‐by‐layer calculation of the diffraction field. Second, they stack the reproduced amplitudes at different depths before computing the loss function. These two functions are independent of the parameters within the CNN that can be optimized by gradients. Therefore, this counterintuitive conclusion can be drawn: the encoding capability of the neural network is closely related to the distributions of intensity and depth but is independent of the correspondence between intensity and depth.

## 3 Virtual Depth Generation for 2D Images Based on Random Mapping and Resampling Method

**Figure 4:**
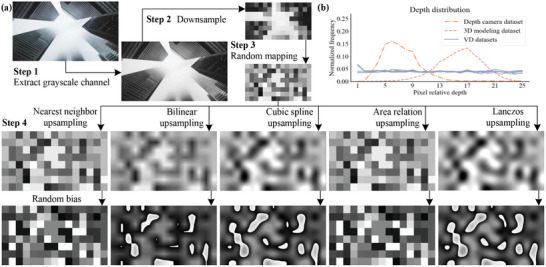
Obtain VD from a RGB image. a) Step 1 involves extracting the grayscale channel, step 2 involves downsampling the image, step 3 involves random mapping, step 4 involves upsampling the mapping values, and step 5 involves applying a random bias, b) the statistical histogram of depth distribution indicates that, relative to the depth camera dataset NYU^[^ ^50^ ^]^ and 3D modeling dataset,^[^ ^18^ ^]^ the VD datasets obtained by different sampling methods in Step 4 exhibit a more uniform pixel distribution across 25 depth layers.

According to the conclusions from the previous chapter, we can assign VD to 2D images through various methods. The resulting depth values should adhere to a distribution rule that ensures all depths are as evenly distributed as possible. Additionally, the depth assignment can incorporate the characteristic of a depth map where the grayscale transitions smoothly from near to far, maintaining a focused boundary. We propose a random mapping and resampling method (RMRM), as illustrated in **Figure** **4**. The core steps of this method are as follows:

**Step 1** involves extracting the grayscale channel. Initially, the color image is converted into a grayscale image. Grayscale image pixel values range from 0 (representing black) to 255 (representing white). Grayscale processing methods employed in this paper is the weighted average grayscale processing. The human eye is least sensitive to the color blue, with the highest sensitivity to green. Therefore, by applying the weighted average recommended by the International Telecommunication Union (ITU‐R) with ratios of 0.299, 0.587, and 0.144 to the RGB intensity image *I*(*x*, *y*), a more reasonable grayscale image *I*_Gray_(*x*, *y*) can be obtained. The calculation process is represented as follows:

(9) $$\begin{array}{ccc} I_{\text{Gray}}\left(x,y\right) & = & 0.299I_{\text{RGB}\_R}\left(x,y\right)+0.587I_{\text{RGB}\_G}\left(x,y\right)\\ & & +\;0.144I_{\text{RGB}\_B}\left(x,y\right) \end{array}$$

where, $I_{\text{RGB}\_R}$, $I_{\text{RGB}\_G}$ and $I_{\text{RGB}\_B}$ denote the red, green, and blue channels of the RGB intensity image *I*_RGB_, respectively.

**Step 2** involves downsampling the image. In order to diminish the details in the original intensity image and blur the intensity information, which is advantageous for subsequent processing, we initially downsample the grayscale image *I*_Gray_(*x*, *y*) to obtain $I_{\text{Dsmp}}\left(\frac{x}{s},\frac{y}{s}\right)$. The calculation process is represented as follows:

(10) $$I_{\text{Dsmp}}\left(\frac{x}{s},\frac{y}{s}\right)=I_{\text{Gray}}\left(x,y\right)$$

where, upsampling is characterized by the reduction factor *s*. Assuming the original intensity image has dimensions (M, N), the size of the subsampled image becomes $\left(\frac{M}{s},\frac{N}{s}\right)$.

**Step 3** involves random mapping. To ensure consistency across multiple depth distributions and an equitable distribution of training samples among different depth neural networks, while maintaining computational efficiency, a series of uniformly distributed thresholds *T*_0_, *T*_1_, · · ·, *T*_*k*_ is employed, derived from the statistical histogram distribution, where *T*_0_ = 0, *T*_*k*_ = 255, *k* denotes the *k*th threshold. The process of randomly mapping pixels within the thresholds can be expressed as:

(11) $$I_{\text{Thsg}}\left(x,y\right)=T_{r},T_{n}\leq I_{\text{Dsmp}}\left(x,y\right)\leq T_{n+1}$$

where, *I*_Thsg_(*x*, *y*) denotes the image obtained by random mapping, with 0 ⩽ *n* ⩽ *k* − 1, *r* is a random integer between 0 and *k*.

**Step 4** involves upsampling the mapping values and applying a random bias. Utilizing interpolation techniques like nearest‐neighbor upsampling, bilinear upsampling and bivariate cubic upsampling, we rescale the segmented image back to its original dimensions. Let *D*(*x*, *y*) denote the VD image obtained after upsampling.

In the case of nearest‐neighbor upsampling, the computation process for the depth image *D*_(0)_(*x*, *y*) is represented as:

(12) $$D_{\left(0\right)}\left(x,y\right)=I_{\text{Thsg}}\left(\text{round}\left(s\cdot x\right),\text{round}\left(s\cdot y\right)\right)+\text{rand}\left(0,255\right)$$

where, *s* represents the reduction factor used in the preceding image downsampling, round(*s* · *x*) is the integer coordinates obtained by rounding *s* · *x*. The function rand(0, 255) returns a randomly chosen integer within [0, 255]. Assuming the dimensions of the image after random mapping are (M, N), the dimensions of the image after upsampling would be (*s* · M, *s* · N).

In the case of bilinear upsampling, the computation process can be expressed as follows:

(13) $$\begin{array}{c} D_{\left(1\right)}\left(x,y\right)=\left(1-\alpha \right)\left(1-\beta \right)I_{\text{Thsg}}\left(\lfloor s\cdot x\rfloor ,\lfloor s\cdot y\rfloor \right)\\ \;\;\;\;\;\;\;\;\;\;+\;\alpha \left(1-\beta \right)I_{\text{Thsg}}\left(\lfloor s\cdot x\rfloor +1,\lfloor s\cdot y\rfloor \right)\\ \;\;\;\;\;\;\;\;\;\;+\;\left(1-\alpha \right)\beta \cdot I_{\text{Thsg}}\left(\lfloor s\cdot x\rfloor ,\lfloor s\cdot y\rfloor +1\right)\\ \;\;\;\;\;\;\;\;\;\;+\;\alpha \cdot \beta \cdot I_{\text{Thsg}}\left(\lfloor s\cdot x\rfloor +1,\lfloor s\cdot y\rfloor +1\right)\\ \;\;\;\;\;\;\;\;\;\;+\;\text{rand}\left(0,255\right) \end{array}$$

where, α = *s* · *x* − ⌊*s* · *x*⌋, and β = *s* · *y* − ⌊*s* · *y*⌋, where ⌊ · ⌋ denotes the floor function.

In the context of bivariate cubic upsampling, the computational process can be formulated as follows:

(14) $$\begin{array}{ccc} D_{\left(2\right)}\left(x,y\right) & = & \sum_{i=0}^{3}\sum_{j=0}^{3}w\left(i-\alpha \right)w\left(\beta -j\right)I_{\text{Thsg}}\\ & & \left(\lfloor s\cdot x\rfloor +i-1,\lfloor s\cdot y\rfloor +j-1\right)+\text{rand}\left(0,255\right) \end{array}$$

The calculation process for the weight factor *w*(*t*) is represented as:

(15) $$w\left(t\right)=\left\{\begin{array}{c} \left(a+2\right){\left|t\right|}^{3}-\left(a+3\right){\left|t\right|}^{2}+1,\\ a{\left|t\right|}^{3}-5a{\left|t\right|}^{2}+8a\left|t\right|-4a,\\ 0, \end{array}\right.\begin{array}{c} \text{if}\;\left|t\right|\leq 1\\ \text{if}\;1<\left|t\right|\leq 2\\ \text{otherwise} \end{array}$$

where, $a=-\frac{1}{2}$.

For area relation upsampling, the effect of image magnification is similar to that of nearest‐neighbor interpolation. For Lanczos upsampling, calculated from adjacent 8 × 8 pixels, the formula is similar to bilinear and bicubic interpolation.

With the above four steps, various depth transition effects from near to far can be simulated. As illustrated in Figure 4, different parameter combinations yield VD generation results, the statistical histogram of depth distribution indicates that, relative to the depth camera dataset and 3D modeling dataset, the VD datasets exhibit a more uniform pixel distribution across 25 depth layers.

In comparison to depth camera and 3D modeling two methods, RMRM, by avoiding time‐consuming iterative computations, can rapidly generate images simulating depth information, achieving every VD within a mere 0.17 s and generating a 4K phase‐only hologram in only 0.061 s, offering economic and time efficiencies over traditional methods. Consequently, this method is particularly suitable for applications that require swiftly obtaining depth image datasets. Furthermore, due to its simplicity in computation, it is also suitable for integration into hardware systems with limited resources.

## 4 Results and Discussion

The computational platform for holographic numerical simulation is constructed using Python 3.9.18, PyTorch version 2.0.1, and CUDA version 11.7. The U‐Net model, specifically designed for this platform, underwent training and testing on the NVIDIA GeForce RTX 3090 Ti GPU, and dataset generation took place on an Intel(R) Core (TM) i7‐8700K 3.70 GHz CPU. We applied the Adam optimizer to optimize weights and biases, initializing the learning rate at 0.001. Every 2000 training iterations, the learning rate was halved. A total of 50 epochs were conducted for each category of the dataset, and each epoch contained 200 pairs of RGB‐depth images.

**Figure 5:**
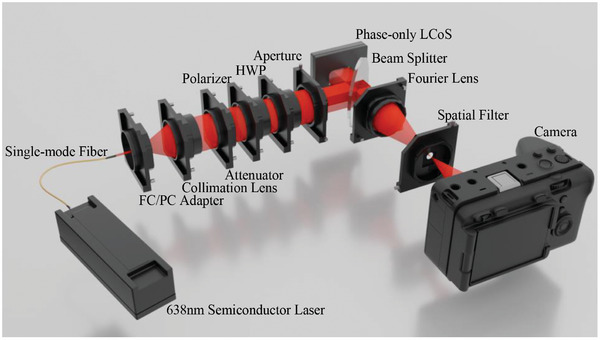
Optical experiment setup. Only the red laser is visualized.

The optical experimental platform utilized in this study is illustrated in **Figure** **5**. The reconstructed light source is a single‐mode optical fiber with a central wavelength of 638 nm, power of 30 mW, and core diameter of 4 µm. Its wider spectral linewidth aids in suppressing coherent noise, thereby improving the quality of reconstructed images. The output end of the single‐mode fiber is placed on the focal plane of a collimating lens with a focal length of 100 mm, serving as a nearby point light source to achieve plane wave illumination. Neutral density filters are used for light attenuation and linear polarization, followed by a half‐wave plate to align the polarization direction with the optimal polarization direction of the LCoS spatial light modulator (CAS Microstar FSLM‐4K70‐P02). The LCoS specifications include a resolution of 4094 × 2400 pixels, pixel pitch of 3.74 µm, and phase‐reflective type. After loading the holographic image, the LCoS modulates the incident laser through phase reflection, achieving the reproduction of a 3D light field with multiple diffraction orders. Specific diffraction orders are selected, passing through a Fourier lens with a focal length of 150 mm to obtain an inverted real image of the target scene. Finally, a bandpass spatial filter is employed to further restrict the diffraction orders that can pass through, filtering out other diffraction orders and additional interferences. The reconstructed 3D scene is directly captured by the lensless Canon EOS 5D Mark III digital camera's CCD. Adjusting the camera's position allows for reproduction of images at different depths.

As the SLM can only modulate monochromatic light, this paper utilizes grayscale image datasets for training and testing neural network models. In the optical experimental setup, red laser light is employed. If the hardware is sufficiently advanced, generating holograms separately for each color channel of the dataset and subsequently reconstructing color images is feasible. Reconstruction of color images can be achieved through time‐division multiplexing and the generation of corresponding holograms for RGB channels. To evaluate the quality of the reconstructed images, we compare the peak signal‐to‐noise ratio (PSNR) and structural similarity index (SSIM) of the original intensity images in the 3D scene with the reconstructed image intensity at different focal planes.

**Figure 6:**
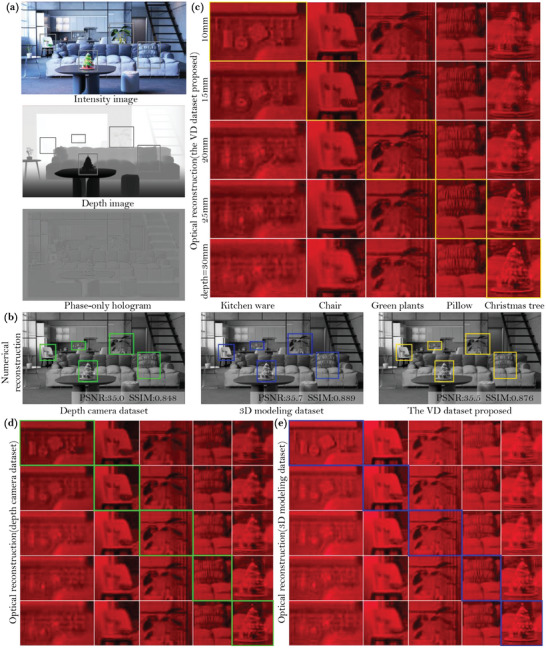
Evaluation of the indoor 3D scene numerical and optical reconstruction quality of CNN models trained on three datasets. a) Intensity image, depth image and phase‐only hologram of the scene obtained by training the model with the VD dataset, b) numerical reconstruction of the target focal stack, (c–e) represent optical reconstructions achieved through phase‐only holograms trained on the VD dataset, depth camera dataset, and 3D modeling dataset, respectively.

To evaluate the effectiveness of the proposed method in handling complex scenes, we used Blender software to create a 3D indoor scene and rendered both intensity and depth images. As illustrated in **Figure** **6**a, we obtained intensity and depth images for the 3D scene, with the depth information categorized into five intervals. Our primary attention was directed toward objects such as kitchen ware, chair, green plants, pillow, and Christmas tree. We computed diffraction fields using three datasets: the depth camera dataset NYU,^[^ ^50^ ^]^ the 3D modeling dataset,^[^ ^18^ ^]^ and our proposed DIV2K VD dataset. The MIT‐CGH‐4K dataset, though commonly employed in holographic research, was not included due to its lower native resolution (384 × 384 and 192 × 192), which is incompatible with our hardware's 2304 × 4094 pixel format. Attempting to upscale or tile the dataset introduces artifacts that significantly degrade the quality of neural network training and holographic rendering. The NYU dataset needed to be converted to 4K size using interpolation methods. These RGB‐Ds were then fed into U‐Net. The resulting hologram is shown in Figure 6a, representing the holographic image generated by the neural network trained on the VD dataset. Subsequently, we obtained a pixel overlay image for the numerical focal stack reproduction of the 3D scene, as depicted in Figure 6b. The PSNR values for holographic images after multi‐depth reproduction using the depth camera dataset, 3D modeling dataset, and VD datasets were 35.0, 35.7, and 35.5 dB, respectively. The SSIM values were 0.848, 0.889, and 0.876, respectively. This indicates the high quality of our proposed dataset. The optical reproduction of various objects of interest is illustrated in Figure 6c–e, revealing that holograms generated by models trained on different datasets can correctly focus during optical reproduction. However, careful observation reveals that the reconstruction quality aligns closely with numerical reproduction. A more detailed magnification of the numerical and optical experimental data reveals significant variations in the detail of kitchenware, items above the chair, and overall image clarity. Readers are encouraged to zoom in on the original images to observe these experimental outcomes more precisely. Demonstrating the numerical and optical reconstruction of this indoor scene hologram representing the holographic image generated by the neural network trained on the VD dataset can be found in Videos S1 and S2 (Supporting Information). The numerical and optical reconstruction of the bunny scene hologram can be found in Supplementary Figure S1 (Supporting Information).

**Figure 7:**
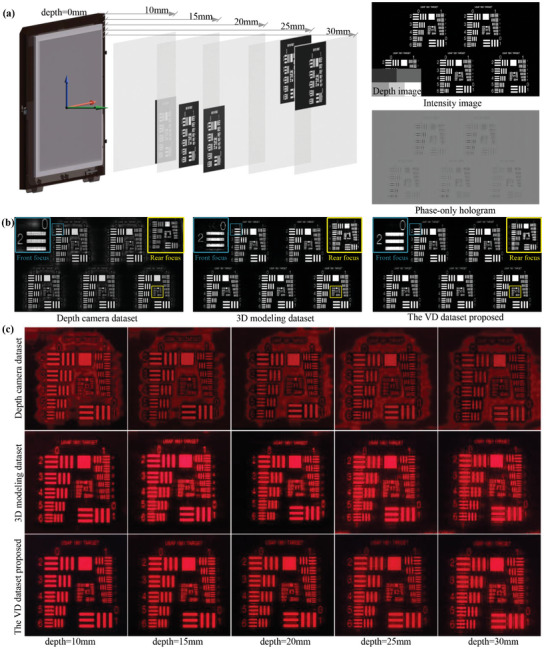
Evaluation of the 3D test pattern scene numerical and optical reconstruction quality of CNN models trained on three datasets. a) Multi‐depth scene, intensity image, depth image and phase‐only hologram of the scene obtained by training the model with the VD dataset, b) numerical reconstruction of the target focal stack, c) represent optical reconstructions achieved through phase‐only holograms trained on the VD dataset, depth camera dataset, and 3D modeling dataset.

**Figure 8:**
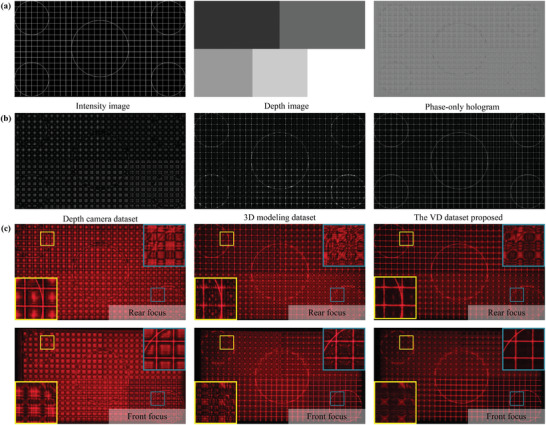
Evaluation of the 3D line test pattern scene numerical and optical reconstruction quality of CNN models trained on three datasets. a) Intensity image, depth image and phase‐only hologram of the scene obtained by training the model with the VD dataset, b) numerical reconstruction of the target focal stack, c) represent optical front and rear focus reconstructions achieved through phase‐only holograms trained on the VD dataset, depth camera dataset, and 3D modeling dataset.

We also configured a 3D test pattern scene. As depicted in **Figure** **7**a and **Figure** **8**a, we assigned the scene with depth in five intervals. We employed the same three datasets, using the same method as in the indoor scene. Figure 7b illustrates the numerical focal stack reconstruction results corresponding to the three datasets. Figures 7c and 8c show the optical reconstruction results. Notably, we observe a stronger discrepancy in image quality between the numerical and optical results in the binary scene than in the natural scene. This suggests that binary test patterns may better highlight differences in holographic reproduction quality across datasets. By observing the image quality, clarity, and minimum speckle at different depths reproduced by the scene for each dataset, we find that our proposed dataset yields the highest image quality, good clarity, and minimal speckle at all depths.

**Figure 9:**
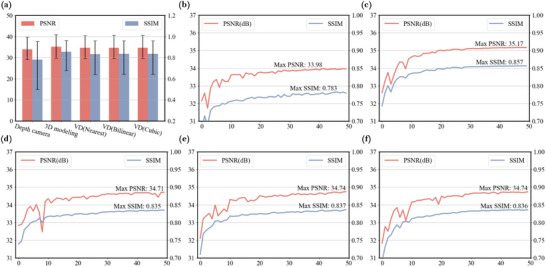
PSNR and SSIM performance of neural network models trained on 3 classes and 5 datasets in the testset(containing 20 RGB‐D of different types of scenes). a) PSNR and SSIM of the model obtained after 50 rounds of training on different datasets on the testset (error bars are taken with 95% confidence intervals), b) and c) showcase line plots of PSNR and SSIM during training on the depth camera dataset and 3D modeling dataset, respectively, d)–f) showcase line plots of PSNR and SSIM during training on the VD dataset acquired via nearest neighbor sampling, bilinear upsampling, and bivariate cubic upsampling, correspondingly.

As shown in **Figure** **9**, our investigation meticulously tracked the neural network training process, comparing the performance across three categories: the depth camera dataset, the 3D modeling dataset, and our proposed VD dataset, utilizing five datasets for assessing the quality of pixel superimposed images in 20 RGB‐D scenes. Statistical results are presented in Figure 9a, PSNR and SSIM of the model obtained after 50 rounds of training on different datasets on the testset(error bars are taken with 95% confidence intervals).

As shown in Figure 9b, the model trained with the depth camera dataset achieved an average PSNR of 34.9 dB and an average SSIM of 0.783 during scene reconstruction. As shown in Figure 9c, the model trained with the 3D modeling dataset obtained an average PSNR of 35.1 dB and an average SSIM of 0.857 in the reconstruction of scenes. As shown in Figure 9d–f, the model trained with our proposed RMRM method, employing the nearest neighbor sampling approach for dataset generation, achieved an average PSNR of 34.7 dB and an average SSIM of 0.835 in scene reconstruction, the model trained with the dataset generated using the bicubic interpolation sampling approach achieved an average PSNR of 34.7 dB and an average SSIM of 0.837 in scene reconstruction, the model trained with the dataset generated using the trilinear interpolation sampling approach achieved an average PSNR of 34.7 dB and an average SSIM of 0.836 in scene reconstruction.

**Figure 10:**
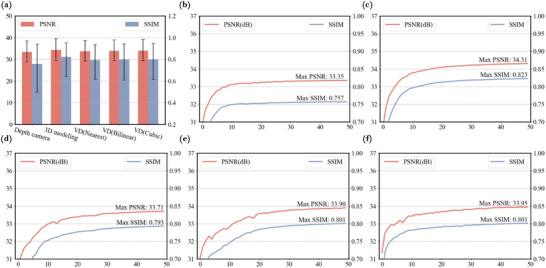
PSNR and SSIM performance of CCNN2‐CGH models trained on 3 classes and 5 datasets in the testset(containing 20 RGB‐D of different types of scenes). a) PSNR and SSIM of the model obtained after 50 rounds of training on different datasets on the testset(error bars are taken with 95% confidence intervals), b) and c) showcase line plots of PSNR and SSIM during training on the depth camera dataset and 3D modeling dataset, respectively, d)–f) showcase line plots of PSNR and SSIM during training on the VD dataset acquired via nearest neighbor sampling, bilinear upsampling, and bivariate cubic upsampling, correspondingly.

To demonstrate the efficacy of the proposed method, we conducted a comparative analysis against the CCNN2‐CGH model in the encoding phase of the CCNN‐CGH model from.^[^ ^47^ ^]^ This comparison, conducted under identical conditions, examined the performance across three categories within five datasets. The average PSNR and SSIM values achieved by our method were slightly lower than those of the U‐Net model, as presented in **Figure** **10**. The experimental results reveal a significant correlation between the quality of multi‐depth holograms produced by neural networks and the quality of the datasets used. This underscores the validity of our proposed 3D dataset‐based holographic encoding solution. Additionally, the findings substantiate a counterintuitive conclusion: the capability of neural networks to encode multi‐depth holograms correlates closely with the intensity and depth distribution in the dataset, rather than any specific intensity‐depth relationship.

## 5 Conclusion 

Experimental results demonstrate a counterintuitive conclusion: the encoding ability of the neural network is closely related to the intensity and depth distributions but is unrelated to the correspondence between intensity and depth. The dataset for the 3D computational holographic neural network provides a nonlinear encoding mapping from complex amplitude to holograms, allowing training without actual depth information. Numerical and optical experiments show that neural networks trained on VD datasets yield reconstructed scenes with high display quality and excellent 3D depth focus. Adjusting parameters such as scaling, thresholds, and interpolation further enhances 3D reconstruction results, often surpassing those from real scene datasets.

This study enables the use of 2D datasets in 3D holography, achieving high‐quality reconstruction by leveraging the diverse and realistic spatial frequency distribution in the DIV2K dataset. This method reduces dataset construction costs, improves model generalization, and allows VD datasets to be expanded and combined with real datasets. The effective utilization of spectral information from 2D datasets in 3D holography enhances model robustness against noise and variations.

Future research will focus on optimizing network structures for more efficient multi‐depth hologram generation and exploring full‐color 3D reconstructions using time‐division multiplexing.

## 6 Evaluation of Reconstruction Quality

PSNR and SSIM are widely employed for assessing the quality of compressed or reconstructed images or video signals. PSNR gauges the similarity between two reconstructed scenes by comparing the peak signal amplitude and noise, while SSIM evaluates their structural information, such as brightness, contrast, and structure. In this study, PSNR and SSIM are employed to assess the reconstruction quality of the target focal stack obtained through multi‐depth reconstruction of in‐focus pixels.^[^ ^36^ ^]^ The calculation processes are respectively represented as:

(16) $$\text{PSNR}=10\cdot \log_{10}\left(\frac{{\text{MAX}}^{2}}{\text{MSE}}\right)$$

where, PSNR is calculated by taking the logarithm (base 10) of the ratio between the square of the maximum possible pixel value, MAX, and the mean squared error (MSE). PSNR is a metric commonly used to quantify the quality of a reconstructed signal by measuring the ratio of the signal strength to the introduced noise. A higher PSNR value indicates better image quality.

(17) $$\text{SSIM}=\left(\frac{2\cdot \mu_{x}\cdot \mu_{y}+C_{1}}{\mu_{x}^{2}+\mu_{y}^{2}+C_{1}}\right)\cdot \left(\frac{2\cdot \sigma_{xy}+C_{2}}{\sigma_{x}^{2}+\sigma_{y}^{2}+C_{2}}\right)$$

where, SSIM is computed using the means(μ_*x*_, μ_*y*_), standard deviations σ_*x*_, σ_*y*_, and cross‐covariance σ_*xy*_ of the compared image patches. The constants *C*_1_ and *C*_2_ are used for stability. SSIM assesses the structural information of two images, including luminance, contrast, and structure. It produces a value between 0 and 1, where 1 indicates perfect similarity.

## Conflict of Interest

The authors declare no conflict of interest.

## Author Contributions

X.Y. and J.L. contributed equally to this work. X.Y. and J.L. conceived and designed the study. X.Y. led the conceptualization, project administration, and supervision. J.L. led the methodology, software development, validation, and visualization. Y.Z. and H.C. contributed equally to investigation, resources, and visualization. H.H. supported formal analysis and writing. T.J. conducted experiments and data analysis. H.L. contributed to data curation, investigation, and writing. Y.Z. contributed to experiments and data analysis. X.B.Y. contributed to conceptualization, project administration, and writing. X.J. performed the data analysis and supervision. All authors contributed to the manuscript and participated in discussions.

## Supporting information

Supplementary Figure

Video S1

Video S2

## Data Availability

The data that support the findings of this study are available from the corresponding author upon reasonable request.
