# Supplementary material for: Generation of Multiple‐Depth 3D Computer‐Generated Holograms from 2D‐Image‐Datasets Trained CNN
Source: Adv Sci (Weinh). 2024 Dec 31;12(8):2408610. doi: 10.1002/advs.202408610 (PMC11848602; doi:10.1002/advs.202408610)
Supplement: Supplementary file 1 — Supplementary Figure [file ADVS-12-2408610-s002.pdf]

ADVANCED SCIENCE
Open Access

Supporting Information

for *Adv. Sci.*, DOI 10.1002/advs.202408610

Generation of Multiple-Depth 3D Computer-Generated Holograms from 2D-Image-Datasets Trained CNN

Xingpeng Yan, Jiaqi Li, Yanan Zhang, Hebin Chang, Hairong Hu, Tao Jing, Hanyu Li, Yang Zhang, Jinhong Xue, Xunbo Yu* and Xiaoyu Jiang*

Supporting Information

Generation of Multiple-Depth 3D Computer-Generated Holograms from 2D-Image-Datasets Trained CNN

Xingpeng Yan, Jiaqi Li, Yanan Zhang, Hebin Chang, Hairong Hu,
Tao Jing, Hanyu Li, Yang Zhang, Jinhong Xue,
Xunbo Yu, Xiaoyu Jiang

Inventory of Supporting Information

- **Supporting Information Figure**

  Supplementary Figure illustrates the numerical and optical reconstruction of the 3D bunny scene generated by CNN models. a) The intensity image, depth image, and phase-only hologram of the scene obtained through training the model with the VD dataset, b)-c) Numerical reconstructions of the target focal stack trained on the VD dataset, d) Optical front and rear focus reconstructions achieved through phase-only holograms, trained on the VD dataset, depth camera dataset, and 3D modeling dataset, respectively.

- **Supporting Information Videos**

  Supplementary Videos 1 and 2 provide the numerical and optical reconstructions of holographic images produced by a neural network

that was trained using the VD dataset, as applied to the indoor scenes described earlier.

The publisher is not responsible for the content or functionality of any supplementary materials provided by the author. For any inquiries (other than those related to missing content), please contact the corresponding author of the article.

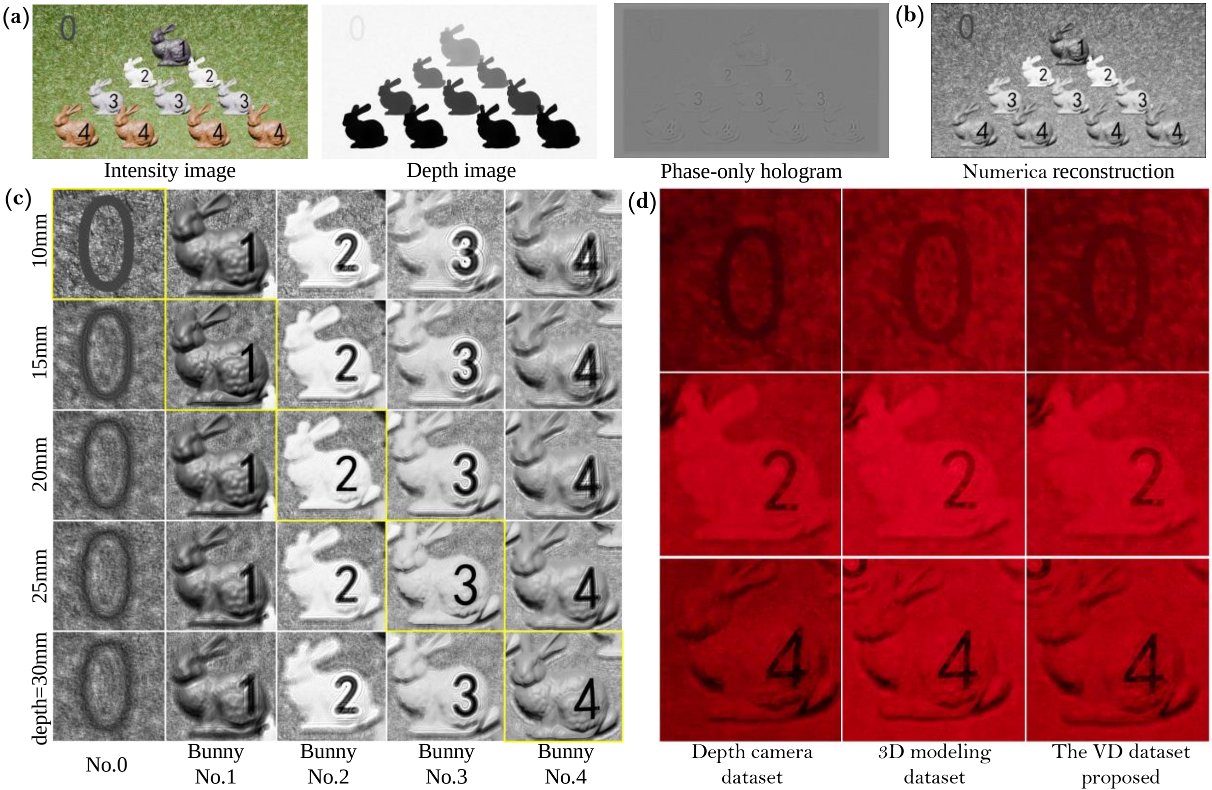

(a)
0
1
2
2
3
3
3
4
4
4
4
Intensity image
Depth image
Phase-only hologram
(b)
Numerica reconstruction
(c)
10mm
15mm
20mm
25mm
depth=30mm
No.0
Bunny No.1
Bunny No.2
Bunny No.3
Bunny No.4
(d)
Depth camera dataset
3D modeling dataset
The VD dataset proposed
